# A Limb-Threatening Long Arterial Dissection Caused by Humerus Neck Fracture: A Case Report

**DOI:** 10.5704/MOJ.1803.012

**Published:** 2018-03

**Authors:** R Kurnaz, M Ikizler, M Ozbayburtlu, T Gunes

**Affiliations:** Department of Orthopaedics and Traumatology, Acibadem Eskisehir Hospital, Eksisehir, Turkey; * Department of Cardiovascular Surgery, Acibadem Eskisehir Hospital, Eksisehir, Turkey

**Keywords:** dissection of axillary artery, humerus neck fracture

## Abstract

Proximal humerus fracture is a common arm trauma and rarely occurs with vascular injury which however is a serious complication. In this case report, we present a long segment dissection of the axillary and brachial arteries as a rare complication due to fragmented proximal humerus fracture and shoulder dislocation. An 80-year old female patient was seen at the emergency department. Radiograph examination has revealed a fragmented proximal humerus fracture besides dislocation of the head of humerus towards the axillary area. On vascular examination, acute arterial occlusion such as absence of radial and ulnar pulses were observed in her left hand. The patient was immediately taken to the operating room. The dissection included the entire segment approximately 20cm between the distal subclavian artery and the distal brachial artery. This injured segment was removed and a 6mm Polytetrafluroethylene (PTFE) graft with rings was interpositoned between subclavian and brachial arteries. This case is a rarity because of such a significant complication after a small injury. Axillary artery injuries caused by humeral neck fractures are rare but should not be missed by the physician.

## Introduction

Proximal humerus fracture is a common limb trauma and rarely occurs with vascular injury which however is a serious complication. This condition has commonly been reported in literature as thrombosis of axillary artery^1^. Some of these reports have also been described as rupture, cut or contusion of axillary artery^2^. In this case report, we present a long segment dissection of axillary and brachial artery as a rare complication due to fragmented proximal humerus fracture and dislocation.

## Case Report

**Fig. 1:**
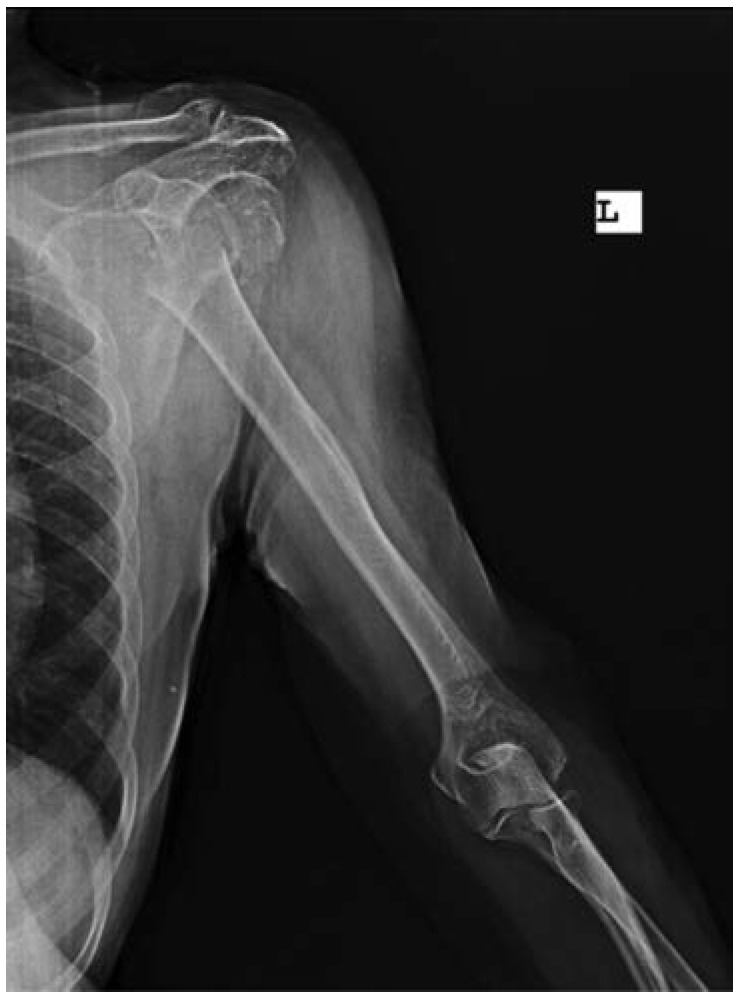
Radiographic image of the humeral head fractures and dislocation.

An 80-year old female patient complained of pain on her left shoulder after her daughter had helped to dress her. As the patient's daughter described it, the arm was into hyper-abduction and external rotation. The patient had diabetes and osteoporosis. The patient was seen at the emergency department after a 6-hour delay. Radiographic examination revealed fragmented proximal humerus fracture besides dislocation of the head of humerus towards the axillary area (Fig. 1).

**Fig. 2:**
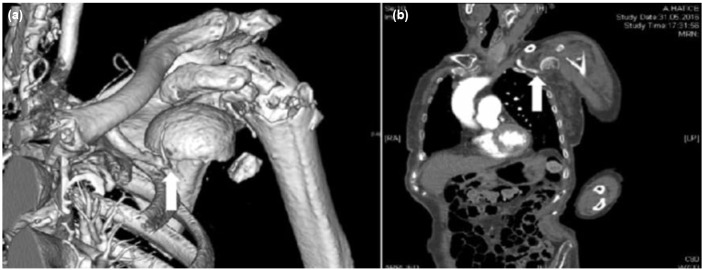
CT angiography of the shoulder (a) 3D Image and (b) Standard Image.

On vascular examination, acute arterial occlusion with absence of radial and ulnar pulses were observed in her left hand. An emergency tomographic angiography was performed and occlusion of the axillary artery was revealed with no circulation to the distal arm (Fig. 2).

The patient was immediately taken to the operating room for vascular surgery and an incision via delta-pectoral approach was performed under general anaesthesia. After exploration, a long segment of arterial dissection was observed in the base of contusion as a consequence of the dislocated humeral head. The dissection included the entire segment approximately 20cm between the distal subclavian artery and the distal brachial artery.

**Fig. 3:**
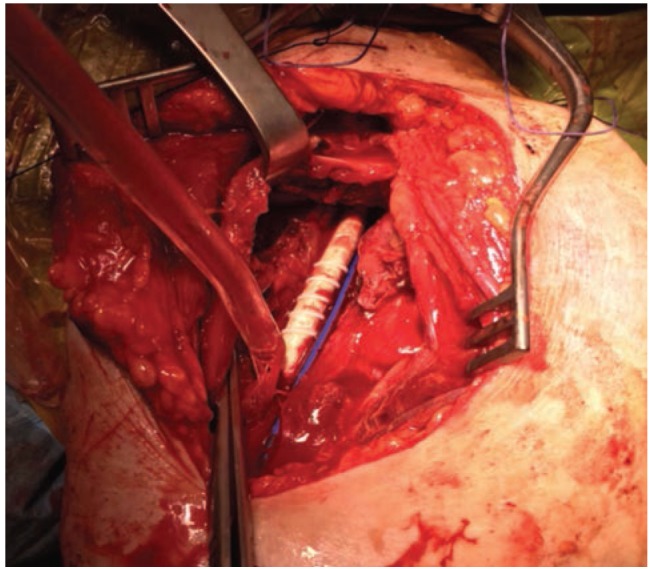
An intra-operative image of the vascular graft *in-situ*.

This injured segment was removed and a 6mm PTFE [Polytetrafluroethylene, Vascutek terumo, Scotland, UK] graft with rings was interpositoned between subclavian and brachial arteries (Fig. 3). After removing the head of humerus, and considering the general condition of the patient, we decided to perform a resection arthroplasty suturing the rotator cuff muscles to the proximal end of the humerus.

The patient was given acetylsalicylic acid 150mg daily for antiaggregant treatment. There were no restrictions on shoulder movements during the postoperative period. After a problem-free post-operative period, the patient was discharged in a healthy condition. In the sixth month postoperative review, she was healthy and well; she could eat without help and the peripheral pulses were palpable in her hand.

## Discussion

Thirty to fifty percent of vascular limb traumas occur in the upper extremities and 5% of them are axillary artery injuries^3^. Most of these are caused by penetrating or cutting injuries. Axillary artery injuries after fractures of the proximal humerus are very rare^4^. Humerus neck fractures are usually caused by hyper-abduction and traction.

The axillary artery is divided into three segments by the pectoralis minor muscle. The first part of the axillary artery lies between the lateral border of the first rib and the medial border of the pectoralis minor muscle. The second part of the axillary artery lies under the pectoralis minor muscle. It gives two branches: the lateral thoracic artery and the thoracoacromial artery. The thoracoacromial artery divides into four terminal branches namely the acromial branch, clavicular branch, deltoid branch and pectoral branch. The third part of axillary artery lies between the lateral border of pectoralis minor and the lower border of the teres major muscle. It gives three branches; the subscapular artery, the anterior circumflex humeral artery and the posterior circumflex humeral artery.

Axillary artery injuries are classified according to these segments. The first segment is the proximal part injury after clavicle fractures. The second segment is a rare injury after humeral neck fractures, which was present in our patient. The third and most common segment injuries are distal artery injuries which represents 89% of the total axillary artery injuries. The number of axillary arterial injuries associated with proximal humerus fractures in the literature is only 12, all of which were third segment injuries. In a case series reported by Ng *et al*, the frequency of first and third segment injuries were equal, and there was no second segment injury^5^.

Vascular injuries such in these traumas are usually seen as rupture, rupture of the intima layer of the artery and thrombosis. According to literature, two out of three patients who were treated with PTFE synthetic graft after an avulsion injury resulted in amputation^4^. As in our case, only three cases with axillary artery dissection were reported in the literature; one of whom was followed up and ended with trans-radial amputation^5^. In case of a prolonged or critical ischemic period, the vascular repair must be performed urgently and prior to the stabilisation of the fracture^4^. In our case, the axillary artery dissection was treated with a PTFE graft and healthy revascularization was achieved despite the presence of such a large segment injury.

Axillary artery injuries caused by humeral neck fractures are rare but should not be missed by the physician. Vascular examinations of patients with such injuries need to be done with attention to details and caution, otherwise they may be missed resulting in amputation of upper extremity. This case is of rarity because of such a significant complicated injury after a trivial trauma. Clearly, the readiness to consider and perform urgent surgical treatment with the combined participation of orthopaedist and vascular surgeons is of paramount importance to ensure the survival of the limb.

## Conflict of Interest

The authors declare no conflicts of interest.
